# Methicillin-Resistant *Staphylococcus aureus* (MRSA) Is Increasing in Norway: A Time Series Analysis of Reported MRSA and Methicillin-Sensitive *S. aureus* Cases, 1997–2010

**DOI:** 10.1371/journal.pone.0070499

**Published:** 2013-08-01

**Authors:** John F. Moxnes, Birgitte Freiesleben de Blasio, Truls Michael Leegaard, Aina E. Fossum Moen

**Affiliations:** 1 Department for Protection, Norwegian Defense Research Establishment, Kjeller, Norway; 2 Department of Infectious Disease Epidemiology, Division of Infectious Disease Control, Norwegian Institute of Public Health, Oslo, Norway; 3 Department of Biostatistics, Institute of Basic Medical Sciences, University of Oslo, Oslo, Norway; 4 Department of Microbiology and Infection Control, Division of Diagnostics and Technology, Akershus University Hospital and Institute of Clinical Medicine, University of Oslo, Lørenskog, Norway; 5 Section of Clinical Molecular Biology and Laboratory Sciences (EpiGen), Division of Medicine, Akershus University Hospital and Institute of Clinical Medicine, University of Oslo, Lørenskog, Norway; Universitätsklinikum Hamburg-Eppendorf, Germany

## Abstract

**Background:**

Accurate estimates of the incidence and prevalence of methicillin-resistant *Staphylococcus aureus* (MRSA) infections are needed to inform public health policies. In Norway, where both MRSA infection and carriage are notifiable conditions, the reported incidence of MRSA is slowly increasing. However, the proportion of MRSA in relation to all *S. aureus* isolates is unknown, making it difficult to determine if the rising incidence is real or an artifact of an increasing number of tests performed.

**Aim:**

To characterize recent trends in MRSA infections and obtain a more complete understanding of the MRSA level in Norway.

**Methods:**

All reported cases of MRSA and methicillin-sensitive *S. aureus* (MSSA) from Oslo County (1997–2010) and Health Region East (2008–2008), representing approximately 11% and 36% of the Norwegian population, respectively, were analyzed using a stochastic time series analysis to characterize trends.

**Results:**

In Oslo County, the proportion of methicillin-resistant cases increased from 0.73% to 3.78% during the study period and was well modeled by an exponential growth with a doubling constant of 5.7 years (95% CI 4.5–7.4 years). In Health Region East, the proportion of MRSA cases increased from 0.4% to 2.1% from 2002 to 2008, with a best-fitting linear increase of 0.26% (95% CI 0.21–0.30%) per year. In both cases, the choice of a linear or exponential model for the time trend produced only marginally different model fits. We found no significant changes due to revised national MRSA guidelines published in June 2009. Significant variations in the increasing time trend were observed in the five hospitals within the region. The yearly reported incidence of MSSA was relatively stable in both study areas although we found seasonal patterns with peaks in August.

**Conclusion:**

The level of MRSA is increasing in Norway, and the proportion of methicillin resistance in all *S. aureus* isolates are higher than the reported proportion of MRSA in invasive infections.

## Introduction

Over the past six decades, bacterial populations have developed resistance to all commercially available agents, and the emergence of antibiotic resistance is considered to be one of the most important threats to human health in the 21^st^ century [1]. *Staphylococcus aureus* (*S. aureus*) is known to quickly develop a resistance to antimicrobial agents. The first penicillin-resistant *S. aureus* was found within a few years of penicillin’s introduction for clinical use, and methicillin-resistant *S. aureus* (MRSA) appeared in 1961, only two years after the introduction of methicillin [2],[3]. Today, MRSA is endemic in hospitals worldwide. In the early 1990s, community-associated MRSA was detected in people lacking the traditional risk factors for MRSA infections; thus, MRSA has become a threat for healthy persons in the general community in addition to the more at risk-population in health-care institutions [4].

The distribution of antibiotic-resistant microbes as well as antibiotic use differs substantially between countries [5]. The annual epidemiological report from the European Centre for Disease Prevention and Control states that the MRSA proportions remain above 25% in more than one-third of the reporting countries, and in the US and the Far East the numbers are even higher [6]. Norway, in addition to other Nordic countries and the Netherlands, has managed to keep the prevalence of MRSA low. Both MRSA infection and carriage are notifiable conditions in Norway. While the number and proportion of MRSA in invasive *S. aureus* infections have been stable at approximately 20 yearly cases and <1%, respectively, the incidence of MRSA isolates has increased in recent years, both in the community and in health-care settings [7]. The increase in MRSA reporting may reflect a true increase in the circulation of MRSA. However, the time trend is difficult to interpret without any knowledge of the total number of tests for *S. aureus* found in cultures during the period. The increase may be biased as a result of intensified testing due to a raised awareness of the problem, underreporting from laboratories prior to the establishment of a national MRSA reference laboratory in 2006, and changes in screening practices at health institutions following the introduction and revision of national guidelines for MRSA infection control in 2004 and 2009, respectively [8], [9].

In this article, we study the MRSA proportion of all *S. aureus* isolates from 1997 through 2010 using time series analysis to give a comprehensive picture of the recent development of methicillin resistance in Norway. Unlike most other statistical methods, time series analysis does not necessarily assume that the data are generated independently, the dispersion may vary with time and the time series may be governed by a trend that could have cyclical components. Some previous studies have used time series analysis to study the evolution of antimicrobial resistance and infection control policies [10]–[18]. In addition, we use interrupted time series analysis to identify the potential effects on MRSA dynamics of a recent revision in the national guidelines for MRSA control in June 2009. The main changes in the updated guidelines were broader and more detailed guidelines for handling MRSA-infected persons in health care services outside of hospitals [8],[9]. Recommendations regarding isolation of nursing home residents were changed from isolation until the resident had been declared MRSA-negative to isolation for a limited time period. More focus on infection control efforts within public areas and residents’ rooms were recommended. Updated guidelines for services provided at home or in medical offices provided a more detailed description of recommended procedures in cases of suspected or confirmed MRSA. Lastly, the updated guidelines give specific recommendations about who should be included in the infection control program.

The results from the present study could provide vital information to public health decision-makers.

## Materials and Methods

### Data

Due to limited data availability, we analyzed two data sets. The first dataset (dataset I) consists of all methicillin-sensitive *S. aureus* (MSSA) and MRSA isolates collected in Oslo County from 1997–2010; the study area includes the Norwegian capital of Oslo and nearby surrounding areas, covering approximately 11% of the Norwegian population. The second dataset (dataset II) consists of all MSSA and MRSA isolates collected in Health Region East from 2002–2008 and 2002–2010, respectively. Health Region East consists of Oslo County and four neighboring counties and is the most populated area of Norway; it includes many large and small cities and rural areas, and covers approximately 36% of the Norwegian population. Use of a shorter time period for MSSA data in the second dataset was necessary because of changes in 2002 and 2008 in the database software by several of the participating microbiological laboratories.

Approximately 30% of the data from Oslo County 1997–2010 was also included in the second dataset.

### Dataset I: MSSA and MRSA Isolates from Oslo County, 1997–2010

Bacterial samples collected from September 1997 through December 2010 were analyzed at the microbiological laboratory at Oslo University Hospital Trust; Ullevål Hospital. MSSA and MRSA isolates were identified using routine diagnostic procedures and registered in the database system, SWISSLAB (Swisslab, GmbH, Berlin, Germany). The data for this study included information on the month/year when the bacterial sample was collected. Repeat MSSA and MRSA isolates from the same individual in the study period were excluded and only the first MSSA and MRSA isolate were included. The authors received anonymized data.

### Dataset II: MSSA and MRSA Isolates from Health Region East, 2002–2010

Bacterial samples collected from January 2002 through March 2010 in Oslo County were analyzed and registered as described above (dataset I), with some exceptions regarding MRSA isolates, described later in this section. During the same time period, bacterial samples collected in Akershus, Oppland, Hedmark and Østfold Counties were analyzed at the microbiological laboratories of Akershus University Hospital Trust, Vestre Viken Hospital Trust; Asker and Bærum Hospital, Innlandet Hospital Trust and Østfold Hospital Trust. MSSA and MRSA isolates were identified using routine diagnostic procedures and registered in the database system, MICLIS (Miclis AS, Lillehammer, Norway), used in all of these hospitals. Information on MSSA isolates were sent to the authors as anonymized data, while MRSA isolates from all the counties and hospital trusts were sent to and registered at Akershus University Hospital Trust. The data for this study included information on the month/year when the bacterial sample was collected. As with dataset I, repeat MSSA and MRSA isolates from the same individual in the study period were excluded and only the first MSSA and MRSA isolates were included.

In dataset II 650 isolates were included from Oslo University Hospital; Ullevål Hospital in the time period 2002–2010, compared to 960 isolates in dataset I. As we allow only one MRSA isolate per person in the present study, a probable reason for this discrepancy is that registration of persons with MRSA isolates at a participating microbiological laboratory occurred prior to registration of the person at Ullevål Hospital. In such instances, the MRSA isolate from Ullevål Hospital will be excluded from dataset II. Another possible explanation is that the data were extracted from two different databases. For dataset I the data were extracted from SWISSLAB at Oslo University Hospital; Ullevål Hospital. For dataset II the data were extracted from the MRSA database at Akershus University Hospital, which had been part of the regional MRSA reference laboratory until 2010.

### Time Series Analysis

In a time series analysis, the usual situation is to have a set of random variables {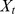}, which could be discrete or continuous, defined for all values of the real number *t* (time). The outcome of a random variable is a state value, while a set of random variables is called a stochastic process, which is completely determined if the joint distribution of the set of random variables 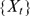 is known. A realization of the stochastic process is an assignment to each *t* in the set of 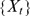, a value of 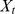. What is essential in stochastic theories is how randomness is accounted for [19].

In Norway the incidence of MRSA is still rather rare. In order to account for such rare events, stochastic theories are important for modelling. We model the monthly reported number of MRSA as a random variable 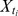, where 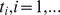, is a variable indicating time in months from start of the study period. For simplicity we write 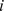 instead of 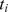 in the formulas.

Likewise, we let the random variable 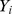 be the monthly reported number of MSSA at time 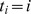. Hence, the variable 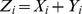 represents the total number of reported *S. aureus* (MRSA+MSSA) cases in month *i* counted from the start of the observation period.

Obviously, the number of positive MRSA tests, 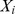, is dependent on the number of *S. aureus* tests, 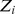. We let the stochastic variable 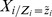 be the number of MRSA at time 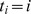 given 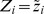, where 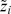 is the actual reported number of *S. aureus* in month 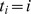. We name the probability that a *S. aureus* positive case is methicillin-resistant, 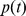. For simplicity we write 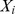 for 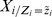.

The strategy for constructing a feasible stochastic process should be based on a three-step iterative cycle. The first step is model identification, the second step is model estimation and the third step concerns diagnostic checks on model adequacy.

#### Model identification

We do not model the monthly aggregated number of tests deliveries since the monthly number of test deliveries from institutions and general practitioners is not known. Instead, we will let the monthly total number of reported *S. aureus* (MRSA+MSSA) be given. Assuming independence between each test, the conditional probability 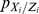
of observing 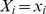 given 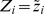 is assumed to be binomially distributed according to:
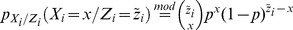
(1)where “mod” refers to the model assumption. We note that in this model 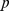 is assumed to depend on time, while being independent of the number of monthly *S. aureus* tests, 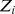. The expectation (mean) and variance is:
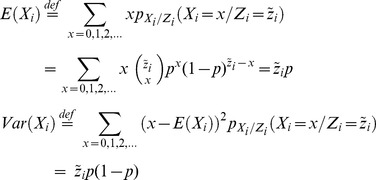
(2)where “def” refers to definition. We let 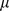 and 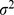
denote the expectation and the variance of the proportion 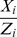 of MRSA. Equation (2) gives that



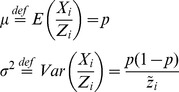
(3)In principle, by studying an infinite number of realizations of 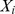 we could determine 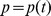, which is simply given by 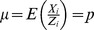. However, we have only one non-stationary time series 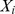 available for use, and in addition, only a finite number of time points. Consequently, we construct an estimator 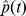 for 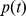 that is as simple as possible, but which still gives a good representation of the available data. There is no unique method for the construction of such an estimator and as a first hypothesis 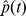 is set deterministic. We apply a least-squares fit (LSF) and compare different functional representations for the time trend of 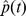: an exponential function, a linear function and a power function. Essentially exponential growth occurs in two different ways: If an entity is self-reproducing, then exponential growth is inherent. If an entity is driven by something else that is growing exponentially, then its growth is derived. However, since 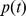 is a probability, it cannot ultimately increase above 1 as time increases. Thus, the increase in 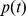 will slow down in a smooth accommodation with its S-shaped growth, although the underlying biological mechanisms for the growth are the same.

Even though we will not model the total number of tests for MRSA+MSSA, it is of some interest to understand the trend 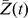 in the total test deliveries. We performed a LSF to the data, and the functional form was based on visual inspection of the data.

#### Model estimation

We apply a LSF of the data of 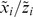 to find the estimator 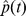. For the proportion of MRSA, we construct the realizations based on the estimator 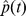:
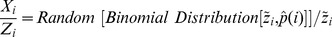
(4)


### Diagnostic Checks on Model Adequacy and Overdispersion

As the first check on the least-squares fit estimator we simulate one time series by considering 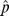 as input. We calculate 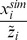 and apply the LSF to find 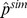, which is compared with 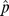. Further, to test model adequacy and dispersion we calculate
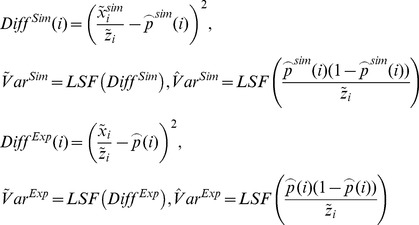
(5)


Good model adequacy gives 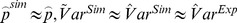. However, overdispersion in the data gives 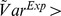 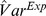. We account for overdispersion by adopting a beta binomial model, which is a common choice for capturing overdisperison in binomial data [20]. This means that we let the probability 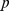 in the binomial distribution be stochastic. The beta distribution is chosen and it has two parameters called 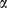 and 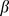 (see for instance http://en.wikipedia.org/wiki/Beta-binomial_distribution). We let 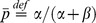. It can be shown that equation (3) becomes
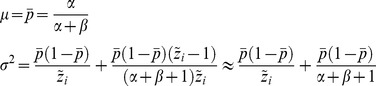
(6)


As a good approximation we set that 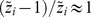. When 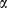 and 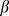 approaches infinity the overdispersion approaches zero. To construct an estimator for 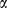 and 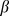 we apply that
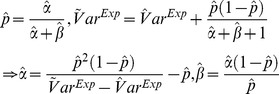
(7)


Thus all together we use three different LSFs to find 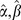 and 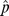. One for 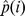 based on the LSF to 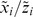, one for 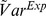 based on the LSF to 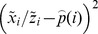 and one for 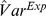 based on the LSF to 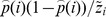.

It is notable that the binomial distribution converges towards the Poisson distribution with 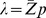 when 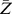 goes to infinity while the product 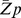 remains fixed. According to two rules of thumb, this approximation is good if 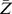 ≥20 and *p*≤0.05, or if 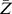 ≥100 and 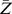 *p*≤10. Our two datasets nearly fulfill the conditions.

The simulations were performed using Mathematica 8 (Wolfram Research Inc., Champaign, IL, USA).

### Ethics Statement

This study has been approved by the Norwegian Regional Committees for Medical and Health Research Ethics, South East, study reference number 2011/2456 C, and by the representative of privacy protection at Akershus University Hospital Trust, study reference number 11/69. The approval from both the Norwegian Regional Committees for Medical and Health Research Ethics, South East, and the representative of privacy protection at Akershus University Hospital Trust, includes the acceptance of using microbiological data from the routine databases in the microbiological laboratories without the need for written consent. Written consent was not needed in the present study as the material used is of microbial origin only and no person identifiable information was gathered. The information gathered from microbial data cannot be traced back to the person from whom it was collected.

## Results

### Dataset I: Oslo County, 1997–2010

#### Trends in identified *S. aureus*

Over the study period from September 1997 through December 2010 (160 months), there were 58,128 MSSA cases and 1,117 MRSA cases identified in Oslo County, with the MRSA cases comprising 1.9% of the total number of *S. aureus* cases. The mean number of monthly *S. aureus* cases exhibited an increasing trend based on a regression of the time series:
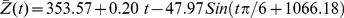
(8)


**Figure 1:**
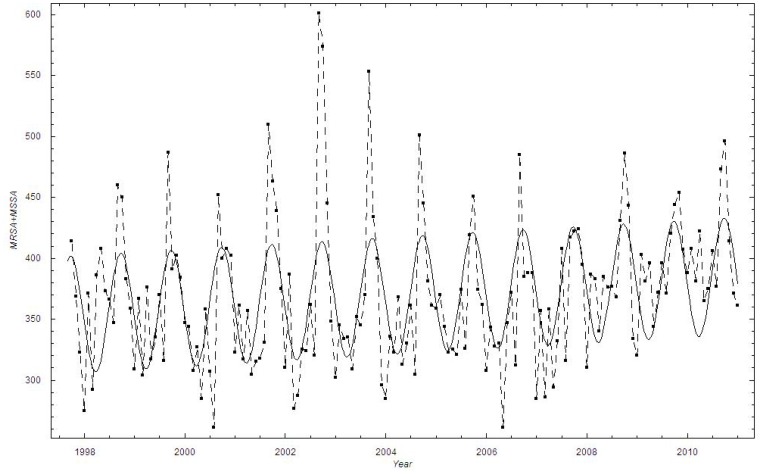
The number of monthly identified *Staphylococcus aureus* cases in Oslo County: September 1997–2010. ▪: Data; A curve showing the estimated mean number of cases 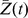 is added.

Overall, the monthly number of identified *S. aureus* cases increased by 9.5% during the study period, from an estimated 353 cases in the autumn of 1997 to 387 cases by the end of 2010, with a yearly average increase of 2.6 cases (Figure 1). The numbers correspond to monthly incidence rates of 71 and 66 per 100,000 individuals in 1997 and 2010, respectively. Noticeably, the data are very scattered with a clear cyclical behavior peaking in the late summer (August). We also performed a discrete Fourier analysis, which confirmed the existence of a seasonal variation in the *S. aureus* data (data not shown).

#### Trends in identified MRSA

**Figure 2:**
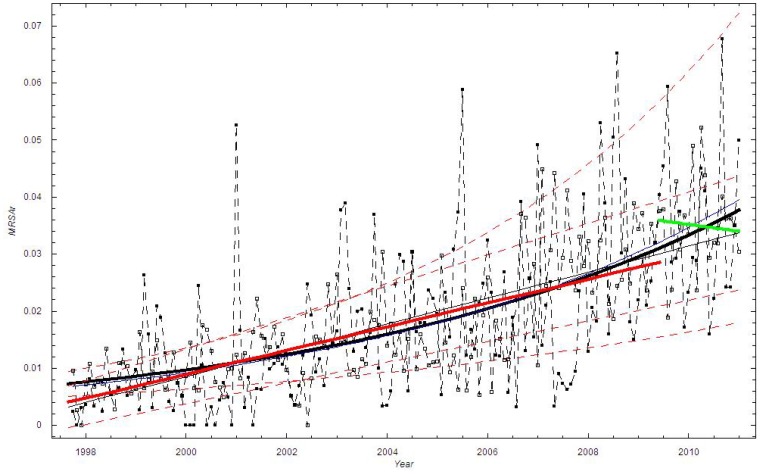
The monthly proportion of MRSA cases in Oslo County: September 1997–2010. Thick black curve: 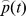 based on exponential LSF; Blue curve: 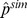; Black curve: 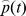 based on linear LSF; Red curve: Linear LSF through June 2009; Green curve: Linear LSF after June 2009; Red dashed curve: 95% confidence bounds on exponential and linear LSF curve; ▪: Data, □: Stochastic simulation (run) 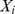.

We estimated the time trend in the mean proportion of MRSA cases using various functional forms (Table 1). The exponential function provided the best fit, closely followed by the linear function. In the exponential model, the proportion of methicillin-resistant cases increased from 0.73% in the autumn of 1997 to 3.78% by the end of 2010, with a doubling constant of approximately 5.7 years (Figure 2).

**Table 1:** Model fit of the proportion of methicillin-resistant *Staphylococcus aureus* for Oslo County and Health Region East to exponential time trend (A), linear time trend (B) and power law time trend (C) with sum of squared errors for three different fit functions and time t in months.

A	Exponential: *a e^b t^*		
	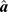 (95% CI)	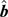 (95% CI)	SSE
Oslo County^1^	0.00732(0.00513–0.00951)	0.0102 (0.00783–0.0127)	0.02147
Health Region East (Tot)^2^	0.00431(0.00281–0.00581)	0.0204(0.0143–0.0264)	0.00197
Akershus University Hospital^2^	0.00979(0.00372–0.0159)	0.0218(0.0111–0.0324	0.0349
Vestre Viken, Asker and Bærum Hospital^2^	0.00356(0.000903–0.00620)	0.0167(0.00326–0.0301)	0.00505
Innlandet Hospital^2^	0.00261(0.000641–0.00459)	0.00646(−0.00901–0.0219)	0.00171
Østfold Hospital^2^	0.00223(0.000227–0.00423)	0.0268(0.0119–0.0417)	0.00519
Oslo University Hospital, Ullevål Hospital^2^	0.00451(0.00189–0.00713)	0.0190(0.00872–0.0292)	0.00556
**B**	**Linear:** *a* + *bt*		
	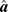 **(95% CI)**	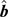 **(95% CI)**	**SSE**
Oslo County^1^	0.00326(−0.000397–0.00692	0.000190(0.000151–0.000230)	0.02149
Health Region East (Tot)^2^	0.00203(−0.000356–0.00443)	0.000216(0.000161–0.000270)	0.001932
Akershus University Hospital^2^	0.000203(−0.00967–0.0101)	0.000636(0.000410–0.000869	0.0329
Vestre Viken, Asker and Bærum Hospital^2^	0.00126(−0.00254–0.00507)	0.000151(0.0000643–0.000238)	0.00489
Innlandet Hospital^2^	0.00268(0.000427–0.00494)	0.0000183(−0.0000332–0.0000670)	0.00172
Østfold Hospital^2^	0.00177(−0.00223–0.00577)	0.000151(0.0000592–0.000241)	0.00540
Oslo University Hospital, Ullevål Hospital^2^	0.00353(−0.000568–0.00762)	0.000174(0.0000799–0.000267)	0.00567
**C**	**Power:** *at^b^*		
	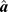 **(95% CI)**	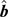 **(95% CI)**	**SSE**
Oslo County^1^	0.000501(−0.0000583–0.00106)	0.827(0.590–1.063)	0.0217
Health Region East (Tot)^2^	0.000568(−0.0000537–0.00119)	0.800(0.523–1.08)	0.00197
Akershus University Hospital^2^	0.000802(−0.000811–0.00241)	0.943(0.439–1.447)	0.0329
Vestre Viken, Asker and Bærum Hospital^2^	0.000526(−0.000702–0.00176)	0.725(0.132–1.317)	0.00486
Innlandet Hospital^2^	0.00214(−0.00121–0.00549)	0.135(−0.297–0.567)	0.00172
Østfold Hospital^2^	0.000203(−0.000385–0.000791)	0.977(0.254–1.700)	0.00546
Oslo University Hospital, Ullevål Hospital^2^	0.00108(−0.000729–0.00289)	0.623(0.194–1.052)	0.00577

1 Dataset I.

2 Dataset II.

The corresponding mean monthly number of MRSA cases in Oslo County was estimated to increase by a factor of 5.4 in the study period, from 2.7 cases (std. 1.0–4.1) in 1997 to 14.5 cases (std. 11.0–18.8) at the end of 2010, thereby representing monthly incidence rates of 0.5 and 2.4 per 100,000 inhabitants in 1997 and 2010, respectively (population statistics from www.ssb.no).

To check whether the revised national guidelines affected the trend, we applied a segmented regression [9]. We employed a linear LSF fit from September 1997 through May 2009, while from June 2009 through December 2010 we performed a new linear LSF. These two lines are shown as the red and the green curves in Figure 2. It appears that the new national guidelines had some impact since the linear trend is decreasing after June 2009, although based on the confidence interval, the time period is too short for this trend to be significant. A visual inspection of Figure 2 suggests that the scatter (dispersion) in the data is larger than in the simulation based on the binomial distribution.

**Figure 3:**
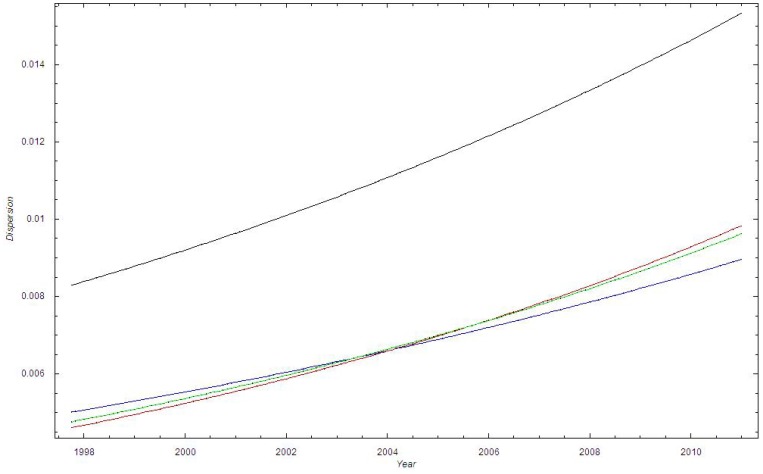
The standard deviation of the proportion of MRSA cases in Oslo County: September 1997–2010. Blue curve: 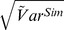; Green curve: 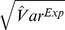; Red curve: 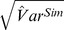; Black curve: 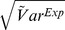
_._

Figure 3 shows that 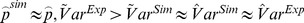. Thus the dispersion of the data is larger than the dispersion in the binomial model with 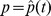.

**Figure 4:**
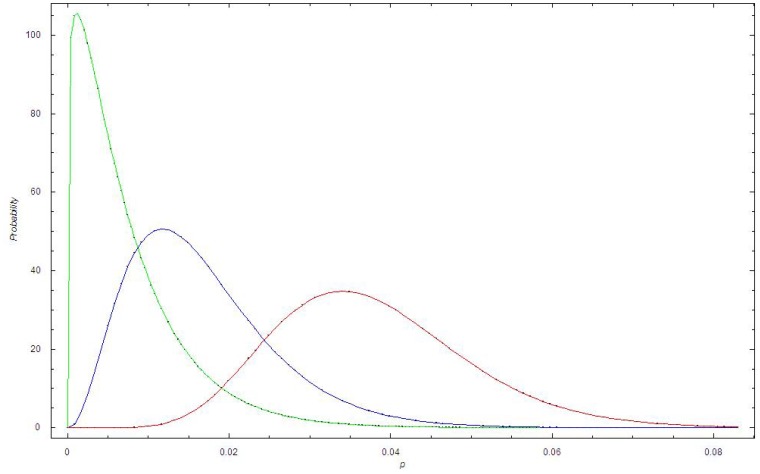
The beta distribution at different times in Dataset I. Green curve: The beta distribution in 1998 for dataset I; Blue curve: The beta distribution in 2005 for dataset I; Red curve: The beta distribution in 2010 for dataset I.

Figure 4 shows the beta distribution for dataset I for 1998, 2006 and 2011. We observe that the beta distribution becomes broader with time and that the expectation shifts to higher values of p.

### Dataset II: Health Region East, 2002–2010

#### Trends in identified *S. aureus*

In the study period from January 2002 through December 2010 (108 months), a total of 1,763 MRSA cases were identified. MSSA data could only be retrieved for the time period from 2002 to March 2008, which yielded a total of 88,106 isolates. We applied a regression for 2002 to March 2008 where both MRSA and MSSA data were available.

The mean monthly number of *S. aureus* cases was weakly declining during the study period based on a linear regression of the time series:
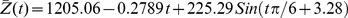
(9)


**Figure 5:**
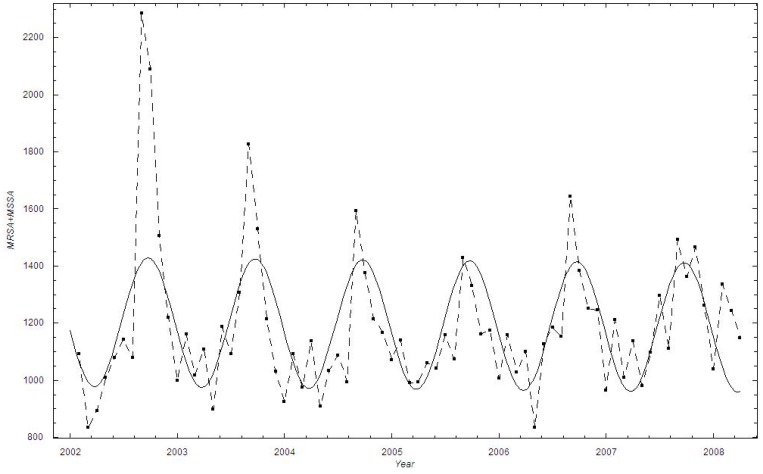
The number of monthly identified *Staphylococcus aureus* cases in Health Region East: 2002–2008. ▪: Data; A curve showing the estimated mean number of cases 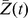 is added.

The numbers declined by 0.2%, from 1187.6 cases in the beginning of 2002 to 1186.3 cases in the spring of 2008 (Figure 5). The numbers correspond to incidence rates of 74 and 67 in 2002 and 2008, respectively. Again, the data exhibit strong cyclical bursting behavior, and are suggestive of seasonal cyclic behavior with peaks around the month of August.

#### Trends in identified MRSA

**Figure 6:**
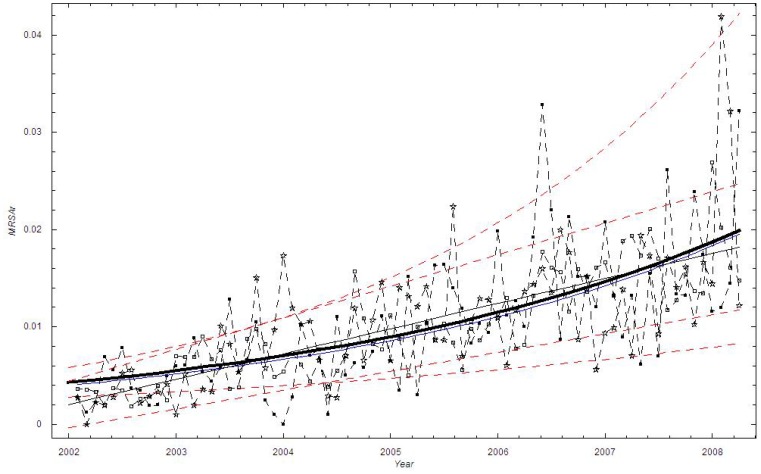
The monthly proportion of MRSA cases in Health Region East: 2002–2008. Thick black curve: 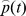 based on exponential LSF; Blue curve: 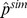; Black curve: 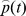 based on linear LSF; Red dashed curve: 95% confidence bounds on exponential and linear LSF curve; ▪: Data, □: Stochastic simulation (run) 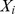,☆: Stochastic simulation based on the beta binomial distribution.

A linear time trend provided the best description of the mean monthly proportion of MRSA cases in Health Region East, and was marginally better than the power function fit and the exponential fit (Table 1). The linear model predicts that the proportion of methicillin-resistant cases increased 0.2% to 1.8% during the six- year study period (Figure 6). Compared with the exponential fit, the linear fit suggests a slightly larger increase during the time period.

**Figure 7:**
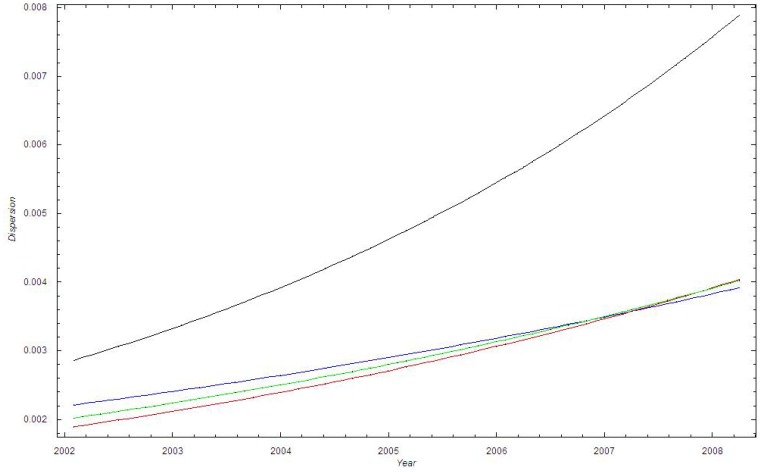
The standard deviation of the proportion of MRSA cases in Health Region East: 2002–2008. Blue curve: 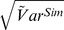; Green curve: 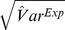; Red curve: 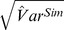; Black curve: 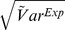
_._

We find good agreement among all the three lowest curves in Figure 7, which shows that 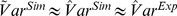. Figure 7 thus shows that the dispersion of the data is larger than the dispersion in the binomial model with 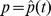.

**Figure 8:**
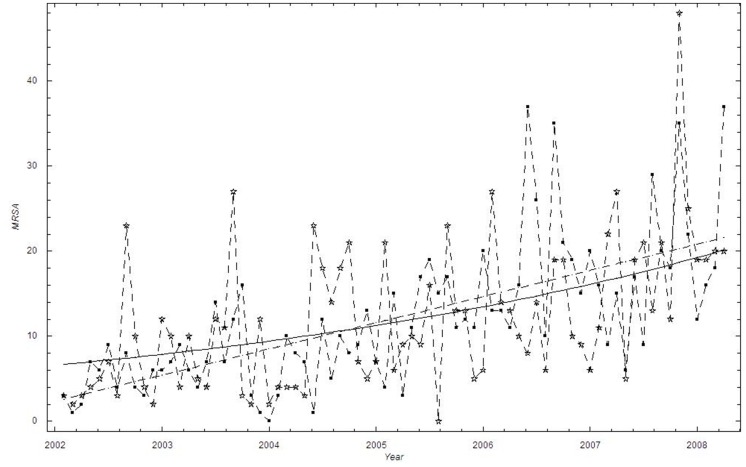
The monthly number of MRSA cases in Health Region East: 2002–2008. ▪: Data, ☆: Stochastic simulation based on the beta binomial distribution. Black curve: Exponential LSF; Black dashed curve: Linear LSF.

The beta distribution for dataset II is very similar to the distribution for dataset I (data not shown). Figure 8 shows the number of MRSA. A trend curve based on a linear and exponential LSF is also shown. Compared with the exponential fit, the linear fit suggests a slightly larger increase during the time period.

As the data were collected from five hospitals, we estimated the time trend in the proportion of MRSA separately for each laboratory (Table 1). The overall conclusion from the analyses is that the model fits using an exponential, a linear or a power law function are only marginally different. Using a linear approximation, the mean yearly increase between 2002 and 2008 varied between 0.02% (95% CI −0.04–0.08%) at Innlandet Hospital to 0.76% (95% CI 0.49%–1.04%) at Akershus University Hospital; both of these estimates are significantly different from the overall yearly trend in Health Region East of 0.26% (95% CI 0.21–0.30%), suggesting a heterogeneous time trend within the region.

## Discussion

To the best of our knowledge, this is the first study to identify the time trend in the proportion of MRSA in *S. aureus* isolates discovered in a large geographical area, regardless of infection type. However, time trends in the proportion of MRSA in *S. aureus* isolates within one or more hospitals have been published [17],[15],[21]. With few exceptions, all bacterial samples in Norway are sent to a medical microbiological laboratory at the regional hospital for testing. In collaboration with the regional hospitals in five counties, we have collected all *S. aureus* findings in the South-Eastern part of the country over long time periods and analyzed these for the presence of MRSA. Our main finding is that the proportion of MRSA has increased in Norway between 1997 and 2010. In Oslo County the increase is well modeled by an exponential growth, with an estimated doubling time of 5.7 years (95% CI 4.6–7.4 years), while in the Health Region East, in the time period 2002–2008, a yearly linear increase by 0.26% (95% CI 0.21–0.30%) gave the best representation of the data. However, in all analyses, the model fits adopting an exponential and linear time trend were only marginally different. At the end of the study periods, the proportion of MRSA cases varied from approximately 2% in Health Region East (2008) to approximately 4% in Oslo County (2010). Both measures are higher compared with the official reported proportion of MRSA in invasive infections in Norway of <1% in 2010 [22]. Our results suggest a steeper growth in the region surrounding the capital compared with the development within the Oslo region. The regional analysis reveals heterogeneities in both the MRSA level and growth rates of the MRSA proportion.

Several studies have shown that increased compliance with infection control measures within hospitals has reduced the MRSA rate as compared to the total *S. aureus* load [23],[15]. We find that despite an increasing awareness and implementation of national guidelines for MRSA control and treatment in the study period, the number of tests for *S. aureus* has somewhat surprisingly decreased by 9–10% when adjusted for population growth. In absolute numbers, the counts of MSSA cases were relatively stable. In Oslo County, the MSSA cases increased by 6.2%, while in Health Region East the number was lowered by 1.6%. The overriding change in the data was caused by the increasing proportion of MRSA cases. Explanations for the increase in the level of methicillin resistance have yet to be investigated, but the *de novo* evolution of MRSA from MSSA, endemic settlements or increased import from abroad could be important reasons. A systematic review from 2011 concluded that MRSA adds to the incidence of MSSA infections rather than replacing them, thus supporting the two latter theories [24]. Moreover, previous studies from our group have revealed heterogeneity in the genetic lineages of the study areas, as well as an increase in a few genetic lineages, hence supporting import and endemic settlements [25],[26].

In 2009, new National MRSA guidelines were launched in Norway [9]. The national MRSA data show a temporarily higher increase in MRSA incidence after the launch, but the incidence returned to a slower rate of increase shortly thereafter [7]. A temporarily higher increase can be expected due to an increased awareness of the bacteria. However, this finding is not reproduced in the present study. We find a non-significant decreasing trend in the dataset from Oslo County using a linear regression after June 2009 (Figure 2). The reasons for the inconsistencies between the present study and the NIPH study could be that the NIPH has national MRSA numbers, while we have studied a region in Norway. In addition, the NIPH only considers MRSA incidence while we show the proportion of MRSA isolates compared to the total *S. aureus* isolates.

The monthly data on identified *S. aureus* cases display seasonality, with peaks around August (cf. Figure 1, Figure 5). The peak in late summer could be the result of people spending more time outside during the warmer months, thereby being more prone to acquiring wound infections. Another explanation could be that many people spend time abroad during summer vacations and those acquiring infections seek medical help once they return home in August. A recent review article comparing studies addressing seasonality in the occurrence of *S. aureus* also concluded that there is an association of warm-weather months with soft-tissue and skin infections caused by *S. aureus* [27].

Based on the binomial distribution (cf. Figure 2, Figure 6), the bursting behavior in the proportion of MRSA cases appeared more irregular throughout the time periods than expected from the binomial stochastic model. We believe that the bursting behavior is primarily related to outbreak investigations, where an MRSA carrier or infected person has been discovered at a health-care institution. When screening is initiated at a nursing home, for example, a large number of persons will be screened, including patients and their relatives, health-care personnel and cleaning staff. However, this will not necessarily increase the proportion of MRSA unless it has a biological basis. It is notable that in Oslo County, several larger and smaller endemic-like outbreaks of MRSA have been documented during the study period [26],[28],[29]. Thus, the bursting behavior is not due to the screening itself, i.e. due to administrative routines, but due to outbreaks in health-care institutions. By applying a beta binomial distribution we were able to account for the additional dispersion (Figure 4).

In the present study we have focused on applying and comparing different functional forms to describe the time trend in the MRSA proportion. This approach is different from the standard time series analysis methodology, in which a time trend parameter is added alongside other potential drivers such as season, hospital and guideline changes, and where the final model is selected based on likelihood ratio tests. We acknowledge that the lack of likelihood based model comparison is a limitation of the present approach. For this reason, we have performed additional analyses with segmented beta-binomial regression models using the Package *betareg* in R [http://cran.r-project.org/web/packages/betareg/] (data not shown). In these analyses we tested the impact of revised national guidelines and within region differences using likelihood ratio tests. These analyses generally confirm the results of the present study, showing a non-significant lowering of the rate of increase following the intervention in 2009. We found significant improvement in the model fit by adding hospital as a fixed effect in the analysis of Health Region East data. The model confirms that there are significant differences in the growth rate across the region with a significantly higher intercept at Akershus University Hospital, and a significantly lower intercept at Vestre Viken, Asker and Bærum Hospital and Innlandet Hospital, compared with Oslo University Hospital, Ullevål Hospital (Results available upon request).

Our study has additional limitations. First, the data were collected from five different hospitals, so differences in methods for retrieving data are possible, although in general the data extraction was quite straightforward. Second, temporary increases in detection and screening could result in more MRSA being identified over shorter periods of time, thereby creating bias in our estimates of proportions over time. Third, to account for the national MRSA policy launched in 2009, we performed an interrupted linear regression of the time trend to study the effect of the intervention However, the one and a half year follow-up period after June 2009 is very short, and more data is needed to achieve conclusive results with respect to the intervention’s impact. Furthermore, the analysis does not take into account any non-linear effects. Last, our model is based on a binomial or beta binomial distribution, with an exponential/linear/power time function for the probability *p(t)* that is estimated by the least-squares fit method. To account for the strong bursting behavior in MRSA, we used a beta-binomial model, but there are other alternative time estimators for *p(t)*. One possibility is to include deterministic low frequency components; yet another possibility would be to apply stochastic differential equations [19]. However, a more realistic model is difficult to construct unless we know more about the biological or administrative reasons for the bursting behavior.

### Conclusions

We find that the proportion of MRSA in relation to the total number of *S. aureus* positive tests is increasing in Norway. In Oslo County a non-significant decrease in the proportion of MRSA is observed following new MRSA guidelines in 2009, warranting further studies.

## References

[1] Cohen ML (1992) Epidemiology of drug resistance: implications for a post-antimicrobial era. Science 257: 1050–1055. 1509255 10.1126/science.257.5073.1050

[2] Shanson DC (1981) Antibiotic-resistant *Staphylococcus aureus* . J Hosp Infect 2: 11–36. 6176621 10.1016/0195-6701(81)90003-7

[3] Jevons MP, Rolinson GN, Knox R (1961) Celbenin-resistant Staphylococci. Br Med J 1: 124–126.

[4] Udo EE, Pearman JW, Grubb WB (1993) Genetic analysis of community isolates of methicillin-resistant *Staphylococcus aureus* in Western Australia. J Hosp Infect 25: 97–108. 7903093 10.1016/0195-6701(93)90100-e

[5] European Centre for Disease Prevention and Control (2010) Antimicrobial resistance surveillance in Europe 2009. Annual report of the European Antimicrobial Resistance Surveillance Network (EARS-Net). Stockholm: ECDC.

[6] Jarvis WR, Jarvis AA, Chinn RY (2012) National prevalence of methicillin-resistant *Staphylococcus aureus* in inpatients at United States health care facilities, 2010. Am J Infect Control 40: 194–200. 22440670 10.1016/j.ajic.2012.02.001

[7] Elstrom P, Kacelnik O, Bruun T, Iversen B, Hauge SH, et al (2012) Meticillin-resistant *Staphylococcus aureus* in Norway, a low-incidence country, 2006–2010. J Hosp Infect 80: 36–40. 22118858 10.1016/j.jhin.2011.10.004

[8] National Institute of Public Health, The Norwegian Directorate of Health (2004) [Infection control 10 MRSA-guidelines]. Oslo: NIPH.

[9] National Institute of Public Health, The Norwegian Directorate of Health (2009) [Infection control 16 MRSA-guidelines]. Oslo: NIPH.

[10] Lopez-Lozano JM, Monnet DL, Yague A, Burgos A, Gonzalo N, et al (2000) Modelling and forecasting antimicrobial resistance and its dynamic relationship to antimicrobial use: a time series analysis. Int J Antimicrob Agents 14: 21–31. 10717497 10.1016/s0924-8579(99)00135-1

[11] Muller AA, Mauny F, Bertin M, Cornette C, Lopez-Lozano JM, et al (2003) Relationship between spread of methicillin-resistant *Staphylococcus aureus* and antimicrobial use in a French university hospital. Clin Infect Dis 36: 971–978. 12684908 10.1086/374221

[12] Vernaz N, Sax H, Pittet D, Bonnabry P, Schrenzel J, et al (2008) Temporal effects of antibiotic use and hand rub consumption on the incidence of MRSA and *Clostridium difficile* . J Antimicrob Chemother 62: 601–607. 18468995 10.1093/jac/dkn199

[13] Aldeyab MA, Monnet DL, Lopez-Lozano JM, Hughes CM, Scott MG, et al (2008) Modelling the impact of antibiotic use and infection control practices on the incidence of hospital-acquired methicillin-resistant *Staphylococcus aureus*: a time-series analysis. J Antimicrob Chemother 62: 593–600. 18467307 10.1093/jac/dkn198

[14] Feng PJI, Kallen AJ, Ellingson K, Muder R, Jain R, et al (2011) Clinical incidence of methicillin-resistant *Staphylococcus aureus* (MRSA) colonization or infection as a proxy measure for MRSA transmission in acute care hospitals. Infect Control Hosp Epidemiol 32: 20–25. 21133793 10.1086/657668

[15] Ellingson K, Muder RR, Jain R, Kleinbaum D, Feng PJI, et al (2011) Sustained reduction in the clinical incidence of methicillin-resistant *Staphylococcus aureus* colonization or infection associated with a multifaceted infection control intervention. Infect Control Hosp Epidemiol 32: 1–8. 21133794 10.1086/657665

[16] Gebski V, Ellingson K, Edwards J, Jernigan J, Kleinbaum D (2012) Modelling interrupted time series to evaluate prevention and control of infection in healthcare. Epidemiol Infect 140: 2131–2141. 22335933 10.1017/S0950268812000179 PMC9152341

[17] Stone SP, Fuller C, Savage J, Cookson B, Hayward A, et al (2012) Evaluation of the national Cleanyourhands campaign to reduce *Staphylococcus aureus* bacteraemia and *Clostridium difficile* infection in hospitals in England and Wales by improved hand hygiene: four year, prospective, ecological, interrupted time series study. BMJ 344: e3005. 22556101 10.1136/bmj.e3005 PMC3343183

[18] Bertrand X, Lopez-Lozano JM, Slekovec C, Thouverez M, Hocquet D, et al (2012) Temporal effects of infection control practices and the use of antibiotics on the incidence of MRSA. J Hosp Infect 82: 164–169. 22980491 10.1016/j.jhin.2012.07.013

[19] Moxnes JF, Hausken K (2010) Introducing randomness into first-order and second-order deterministic differential equations. Adv Math Phys 1–42. DOI: 10.1155/2010/509326.

[20] Hoef JMV, Boveng PL (2007) Quasi-poisson vs. negative binomial regression: How should we model overdispersed count data? Ecology 88: 2766–2772. 18051645 10.1890/07-0043.1

[21] Parienti JJ, Cattoir V, Thibon P, Lebouvier G, Verdon R, et al (2011) Hospital-wide modification of fluoroquinolone policy and meticillin-resistant *Staphylococcus aureus* rates: a 10-year interrupted time-series analysis. J Hosp Infect 78: 118–122. 21497946 10.1016/j.jhin.2011.03.008

[22] European Centre for Disease Prevention and Control (2010) European Centre for Disease Prevention and Control. Annual epidemiological report on communicable diseases in Europe 2010. Stockholm: ECDC. 22114980

[23] Lafaurie M, Porcher R, Donay JL, Touratier S, Molina JM (2012) Reduction of fluoroquinolone use is associated with a decrease in methicillin-resistant *Staphylococcus aureus* and fluoroquinolone-resistant *Pseudomonas aeruginosa* isolation rates: a 10 year study. J Antimicrob Chemother 67: 1010–1015. 22240401 10.1093/jac/dkr555

[24] Mostofsky E, Lipsitch M, Regev-Yochay G (2011) Is methicillin-resistant *Staphylococcus aureus* replacing methicillin-susceptible *S. aureus*? J Antimicrob Chemother 66: 2199–2214. 21737459 10.1093/jac/dkr278 PMC3172038

[25] Fossum AE, Bukholm G (2006) Increased incidence of methicillin-resistant *Staphylococcus aureus* ST80, novel ST125 and SCC*mec*IV in the south-eastern part of Norway during a 12-year period. Clin Microbiol Infect 12: 627–633. 16774558 10.1111/j.1469-0691.2006.01467.x

[26] Moen AEF, Storla DG, Bukholm G (2010) Distribution of methicillin-resistant *Staphylococcus aureus* in a low-prevalence area. FEMS Immunol Med Microbiol 58: 374–380. 20459509 10.1111/j.1574-695X.2009.00649.x

[27] Leekha S, Diekema DJ, Perencevich EN (2012) Seasonality of staphylococcal infections. Clin Microbiol Infect 18: 927–933. 22958212 10.1111/j.1469-0691.2012.03955.x

[28] Leendert van der Werff HF, Steen TW, Garder KM, Andersen BM, Rasch M, et al (2008) [An outbreak of MRSA in a nursing home in Oslo]. Tidsskr Nor Laegeforen 128: 2734–2737. 19079422

[29] Andersen BM, Rasch M, Syversen G (2007) Is an increase of MRSA in Oslo, Norway, associated with changed infection control policy? J Infect 55: 531–538. 18029021 10.1016/j.jinf.2007.09.008

